# Post‐COVID syndrome symptoms, functional disability, and clinical severity phenotypes in hospitalized and nonhospitalized individuals: A cross‐sectional evaluation from a community COVID rehabilitation service

**DOI:** 10.1002/jmv.27456

**Published:** 2021-11-23

**Authors:** Manoj Sivan, Amy Parkin, Sophie Makower, Darren C. Greenwood

**Affiliations:** ^1^ Academic Department of Rehabilitation Medicine University of Leeds Leeds UK; ^2^ Covid rehabilitation service, Leeds Community Healthcare NHS Trust Leeds UK; ^3^ National Demonstration Centre of Rehabilitation Medicine, Leeds Teaching Hospitals NHS Trust Leeds UK; ^4^ Department of Occupational Therapy Leeds Teaching Hospitals NHS Trust; ^5^ Leeds Institute of Cardiovascular and Metabolic Medicine, School of Medicine University of Leeds Leeds UK; ^6^ Leeds Institute for Data Analytics, School of Medicine University of Leeds Leeds UK

**Keywords:** C19‐YRS, long COVID, phenotypes, post‐COVID‐19 condition, SARS CoV‐2

## Abstract

There is currently limited information on clinical severity phenotypes of symptoms and functional disability in post‐coronavirus disease 2019 (COVID) Syndrome (PCS). A purposive sample of 370 PCS patients from a dedicated community COVID‐19 rehabilitation service was assessed using the COVID‐19 Yorkshire Rehabilitation Scale where each symptom or functional difficulty was scored on a 0–10 Likert scale and also compared with before infection. Phenotypes based on symptom severity were extracted to identify any noticeable patterns. The correlation between symptom severity, functional disability, and overall health was explored. The mean age was 47 years, with 237 (64%) females. The median duration of symptoms was 211 days (interquartile range 143–353). Symptoms and functional difficulties increased substantially when compared to before infection. Three distinct severity phenotypes of mild (*n* = 90), moderate (*n* = 186), and severe (*n* = 94) were identified where the severity of individual symptoms was of similar severity within each phenotype. Symptom scores were strongly positively correlated with functional difficulty scores (0.7, 0.6–0.7) and moderately negatively correlated with overall health (−0.4, −0.3, to −0.5). This is the first study reporting on severity phenotypes in a largely nonhospitalized PCS cohort. Severity phenotypes might help stratify patients for targeted interventions and planning of care pathways.

## 1 BACKGROUND

Post‐Coronavirus disease 2019 (COVID‐19) Syndrome (PCS) refers to persistent symptoms 12 weeks after contracting COVID‐19 illness.^1^ There are an estimated more than a million cases of PCS in the UK alone and more than 20 million cases worldwide.^2^ More than 200 symptoms across 10 organ systems have been reported with the most common symptoms being fatigue, pain, breathlessness, palpitations, dizziness, brain fog (cognitive problems), anxiety, depression, posttraumatic stress, skin rash, and allergic reactions^3^ It is a remitting and relapsing condition with a protracted course causing significant distress and disability to the individual.^4^

The symptoms and impact of PCS have been recorded in a variety of ways, including the COVID Symptom Study digital application^5^ and recently developed World Health Organisation clinical platform Case Report Form (CRF).^6^ The Covid‐19 Yorkshire Rehabilitation Scale (C19‐YRS) was the literature's first published and validated patient‐reported outcome measure (PROM).^7^ It was developed by our research team during the first wave of the pandemic ^8^, ^9^ and has been recommended by NICE and NHS England to be used as an outcome measure at first assessment, 6 weeks and 6 months.^10^, ^11^ The scale has been used in several PCS studies supporting the content validity of the scale to reliably capture persistent symptoms.^12^, ^13^, ^14^ Initial psychometric analysis of the scale showed high internal consistency (Cronbach's alpha = 0.89) and good concordance between the overall perception of health and patients' reports of symptoms, functioning, and disability.^7^ The items of the scale span all aspects of 2001 WHO International Classification of Functioning, Disability, and Health (ICF) framework.^8^

Clinical symptom clusters or phenotypes have been mentioned in the literature and some studies have tried to identify these clusters. The Real‐Time Assessment of Community Transmission (REACT) study analyzed more than half a million community cases and identified two symptom clusters – a large cohort with fatigue symptoms and a smaller cohort with respiratory symptoms.^2^ The Post‐hospitalization COVID‐19 study (PHOSP‐COVID) involving 1077 patients did not reveal symptom‐based phenotypes but identified four clusters of posthospitalization outcomes with varying severities of mental and physical health impairment (mild/moderate/severe/very severe) with participants typically reporting nine persistent symptoms five months after discharge.^15^

With the growing number of cases of PCS, there is a need to understand symptom clusters, symptom severity, and interference with daily functioning. By stratifying patients based on severity, appropriate interventions and treatment plans can be prescribed, and the trajectory of the condition mapped. The aim of this study was to explore the presence of symptoms severity phenotypes in a community PCS cohort, including a large proportion of nonhospitalized participants, and understand the relationship between severity of symptoms, functional disability, and overall health in PCS.

## 2 METHODS

### 2.1 Setting

This service evaluation study was conducted within a COVID Rehabilitation service in the North of England. This is a multidisciplinary rehabilitation service, which offers specialist assessment and treatment for PCS, delivered virtually and/or in person, delivered by a specialist multidisciplinary team. Patients are referred via General Practitioners (GPs) when symptoms have persisted beyond 12 weeks and cannot be explained through alternative medical assessment. Patients were not required to have had a positive polymerase chain reaction (PCR) test or antibody test as these were not widely available to the general population of the UK at the start of the pandemic.^16^

### 2.2 Participant identification

Consecutive patients referred to the COVID Rehabilitation service between February 2 and May 3, 2021 were considered for this service evaluation study regardless of whether they had been previously hospitalized with COVID‐19. All patients had agreed for their data to be used anonymously for research purposes or service evaluation. The eligible patients were prompted to complete the latest self‐report version of C19‐YRS by one of the research team members. In total, responses were received from 370 patients.

### 2.3 C19‐YRS

The information gathered on C19‐YRS includes demographic information, medical history, and 16 key symptoms of PCS (including breathlessness, persistent cough, fatigue, pain or discomfort, cognitive problems, anxiety, depression, symptoms of posttraumatic stress disorder [PTSD], palpitations, dizziness, weakness, and sleep problems) and their impact on five daily functions (including communication, mobility, personal care, wider activities of daily living, and social functioning).^8^ Each symptom or functional ability is rated by the respondent on a scale from 0 to 10 (0 being no presence of symptom and 10 being most severe and life disturbing). Overall health status is also captured on a 0–10 numerical rating scale (NRS) scale. Unique to the C19YRS and this study, respondents are also asked to grade their pre‐illness symptoms, functional abilities, and overall health to provide a general clinical baseline for comparison.

### 2.4 Statistical analysis

Overall severity of the 12 most reported symptoms was defined as the mean symptom score, with a mean score of 6 or more considered “severe,” 3 to 5.9 “moderate”, and less than 3 “mild.” Demographic details and brief clinical history were summarized overall and by symptom severity. Functional abilities were reported on the same 0–10 scale.

All pairwise Spearman's correlations across symptoms and functional abilities were calculated and presented graphically as a heat map. Symptoms were then categorized as severe (6 or more) or not, and the frequency of each combination of multiple severe symptoms was presented as an UpSet diagram.

Cluster analysis was used to identify any groupings or co‐occurrence of symptoms that could indicate potential different phenotypes amongst the participants. Two approaches were used: k‐means partition cluster analysis and a hierarchical agglomerative cluster analysis using average‐linkage between clusters and the Euclidian dissimilarity measure. Robustness of results was assessed using 2, 3, and 4 clusters and with different starting values (k‐means cluster analysis) and using different weights and dissimilarity measures (hierarchical cluster analysis). All statistical analyses were carried out using Stata version 16.1 and R version 4.0.5.

### 2.5 Ethical approval

Data were collected in the service as part of routine clinical evaluation and ethical approval was obtained for the secondary analysis of anonymized data collected for the primary clinical purpose which has been completed. A favorable ethical opinion was received from the University of Leeds School of Medicine Research Ethics Committee (Ref MREC 20‐041).

## 3 RESULTS

The majority of participants had not needed any hospitalization during the infection (*n* = 304). The demographic measures and the medical histories for all 370 patients are presented in Table 1. Patients had been experiencing persistent symptoms for a median of 211 days (interquartile range [IQR] 143–353) at the time of completing the C19‐YRS questionnaire. 237 (64%) of the patients were female. The mean age was 47 years (*SD* = 14), with a mean bodyweight of 82 kg (*SD* = 22) and a mean body mass index (BMI) of 29 kg/m^2^ (*SD* = 8).

**Table 1:** Demographic measures and medical history of study participants by hospitalization status

	All*	Not hospitalized	Hospitalized
	(*n* = 370)	(*n* = 304)	(*n* = 66)
Female (%)	237 (64%)	208 (68%)	29 (44%)
Mean age (years) (*SD*)	47 (14)	46 (13)	53 (14)
Mean weight (kg) (*SD*)	82 (22)	80 (21)	93 (22)
Mean BMI (kg/m^2^) (*SD*)	29 (8)	28 (8)	32 (7)
Ethnicity (%)			
White	307 (84%)	256 (86%)	51 (78%)
Black	10 (3%)	6 (2%)	6 (3%)
Asian	40 (11%)	30 (10%)	10 (15%)
Mixed/Other	7 (2%)	7 (2%)	0 (0%)
Smoking status (%)			
Never smoked	235 (65%)	199 (67%)	36 (55%)
Current smoker	24 (7%)	22 (7%)	2 (3%)
Ex‐smoker	105 (29%)	78 (26%)	27 (42%)
Employment status (%)			
Still employed/student	176 (49%)	155 (52%)	21 (33%)
Still retired/homemaker/unemployed	41 (11%)	34 (11%)	7 (11%)
Reduced hours	48 (13%)	44 (15%)	4 (6%)
Sick leave	77 (21%)	48 (16%)	29 (45%)
Stopped work	19 (5%)	16 (5%)	3 (5%)
Date of infection (%)			
UK Wave 1 (March 2020–August 2020)	145 (39%)	128 (42%)	17 (26%)
UK Wave 2a (September 2020–November 2020)	120 (32%)	99 (33%)	21 (32%)
UK Wave 2b (December 2020–May 2021)	88 (24%)	61 (20%)	27 (41%)
UK Wave 3 (June 2021 onwards)	17 (5%)	16 (5%)	1 (2%)
Positive COVID‐19 test (%)	228 (62%)	182 (60%)	46 (70%)
Admitted to hospital (%)	66 (18%)	0 (0%)	66 (100%)
Median duration of symptoms (days) (IQR)	211 (143–353)	223 (150–355)	159 (129–288)

Abbreviations: BMI, body mass index; COVID‐19, coronavirus disease 2019; IQR, interquartile range; *SD*, standard deviation.

* Where numbers do not total 370, this is due to missing data.

Patients were predominantly of white ethnicity (84%) with only 57 patients (16%) from black, Asian, other, or mixed ethnic groups, which is slightly lower than the proportion in the general population of the region served (https://observatory.leeds.gov.uk/population/). Patients from black and particularly Asian ethnic groups reported experiencing more severe symptoms than patients from white ethnic groups.

Half (49%) of patients were still employed or studying on the same hours as before infection with COVID‐19, and 41 (11%) were in the same role as homemakers, still on maternity leave, retired or unemployed. However, 144 (40%) had reduced their work hours, were still on sick leave, or had stopped work altogether because of ill health.

Table 2 shows the three severity phenotypes identified based on their mean symptom score (6 or more considered “severe,” 3–5.9 “moderate”, and less than 3 “mild” as recommended by the C19‐YRS scale). There was a tendency for patients with the most severe symptoms to be more likely female, older, with higher weight or BMI. Those with the most severe symptoms were half as likely to remain employed on the same hours as those with the least severe symptoms.

**Table 2:** Demographic measures and medical history of study participants by overall symptom severity

	All*	Mild	Moderate	Severe
	(*n* = 370)	(*n* = 90)	(*n* = 186)	(*n* = 94)
Female (%)	237 (64%)	48 (53%)	125 (67%)	64 (68%)
Mean age (years) (*SD*)	47 (14)	47 (14)	47 (14)	50 (12)
Mean weight (kg) (*SD*)	82 (22)	79 (19)	81 (20)	88 (27)
Mean BMI (kg/m^2^) (*SD*)	29 (8)	27 (5)	28 (6)	32 (12)
Ethnicity (%)				
White	307 (84%)	79 (88%)	156 (87%)	72 (77%)
Black	10 (3%)	1 (1%)	6 (3%)	3 (3%)
Asian	40 (11%)	5 (6%)	16 (9%)	19 (20%)
Mixed/Other	7 (2%)	5 (6%)	2 (1%)	0 (0%)
Smoking status (%)				
Never smoked	235 (65%)	58 (65%)	120 (66%)	57 (62%)
Current smoker	24 (7%)	3 (3%)	13 (7%)	8 (9%)
Ex‐smoker	105 (29%)	28 (31%)	50 (27%)	27 (29%)
Employment status (%)				
Still employed/student	176 (49%)	57 (66%)	87 (47%)	32 (35%)
Still retired/homemaker/unemployed	41 (11%)	11 (13%)	17 (9%)	13 (14%)
Reduced hours	48 (13%)	8 (9%)	31 (17%)	9 (10%)
Sick leave	77 (21%)	10 (12%)	35 (19%)	32 (35%)
Stopped work	19 (5%)	0 (0%)	14 (8%)	5 (5%)
Date of infection (%)				
UK Wave 1 (March 2020–August 2020)	145 (39%)	36 (40%)	71 (38%)	38 (40%)
UK Wave 2a (September 2020–November 2020)	120 (32%)	31 (34%)	65 (35%)	24 (26%)
UK Wave 2b (December 2020–May 2021)	88 (24%)	20 (22%)	42 (23%)	26 (30%)
UK Wave 3 (June 2021 onwards)	17 (5%)	3 (3%)	8 (4%)	6 (6%)
Positive COVID‐19 test (%)	228 (62%)	49 (54%)	122 (66%)	57 (61%)
Admitted to hospital (%)	66 (18%)	17 (19%)	25 (13%)	24 (26%)
Median duration of symptoms (days) (IQR)	211 (143–353)	211 (144–359)	196 (142–354)	223 (145–346)

Abbreviations: BMI, body mass index; COVID‐19, coronavirus disease 2019; IQR, interquartile range; *SD*, standard deviation.

* Where numbers do not total 370, this is due to missing data.

Radar plot mapping of the average individual symptom score for each of the three categories (Figure 1) showed there was a gradient in all 12 mean symptom scores, with patients who had greater overall symptoms having each separate symptom higher on average with no single symptom driving this association. For patients with milder overall severity scores, fatigue was, on average, the dominant symptom but all mean symptom scores were lower than those deemed to have moderate severity overall. For patients with greater overall severity scores, fatigue was joined by high mean symptom scores across all 12 symptoms recorded, with all mean scores higher than for the moderate category. There was a similar gradient in functional difficulties, depending on the severity of the symptoms (Figure 2). Hospitalized PCS patients reported similar severity levels of persistent symptoms and functional abilities as nonhospitalized patients

**Figure 1:**
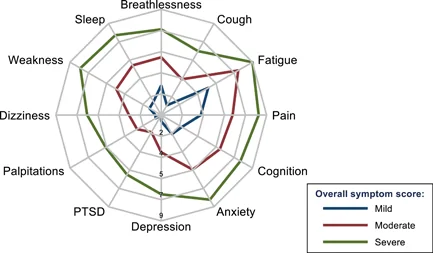
Radar plot of mean severity of 12 persistent long‐COVID symptoms, scored from 0 to 10, by overall severity of the condition. COVID, coronavirus disease

**Figure 2:**
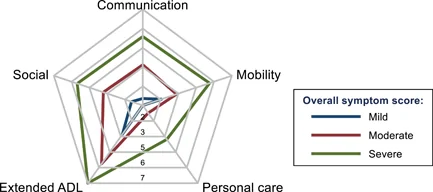
Radar plot of mean severity of 5 functional difficulties, scored from 0 to 10, by overall severity of the condition

Fatigue was the most common symptom experienced, with 353 (95%) reporting this to some extent, followed by 334 (90%) reporting anxiety, 329 (89%) some pain or discomfort, 316 (85%) some breathlessness, and 315 (85%) some cognitive difficulties. Overall symptom scores were negatively correlated with perceived overall health (−0.4, −0.3 to −0.5) and positively correlated with overall functional difficulty scores (0.7, 0.6–0.7). Figure 3 shows a heat plot of the pairwise Spearman's correlation coefficients between each of the core PCS symptoms and associated functional difficulties in the cohort.

**Figure 3:**
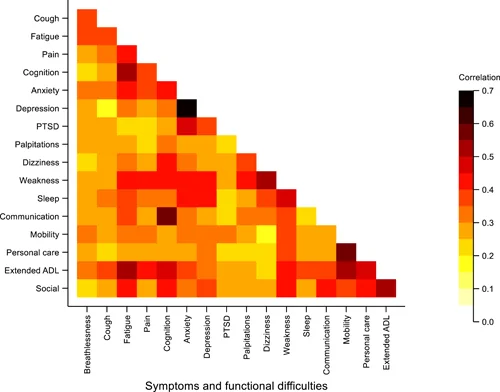
Heat plot displaying the pairwise correlation between core symptoms and functional difficulties. The color gradient reflects the strength of the correlation, with the darker colors indicating stronger correlation

The UpSet diagram in Figure 4 indicates that fatigue and pain or discomfort were the symptoms most frequently experienced as severe. These were experienced across the majority of symptom combinations, with few combinations of severe symptoms that were not combined with pain or fatigue, or both. There were no clear subsets of mutually exclusive symptom combinations that would indicate distinct symptom‐type phenotypes.

**Figure 4:**
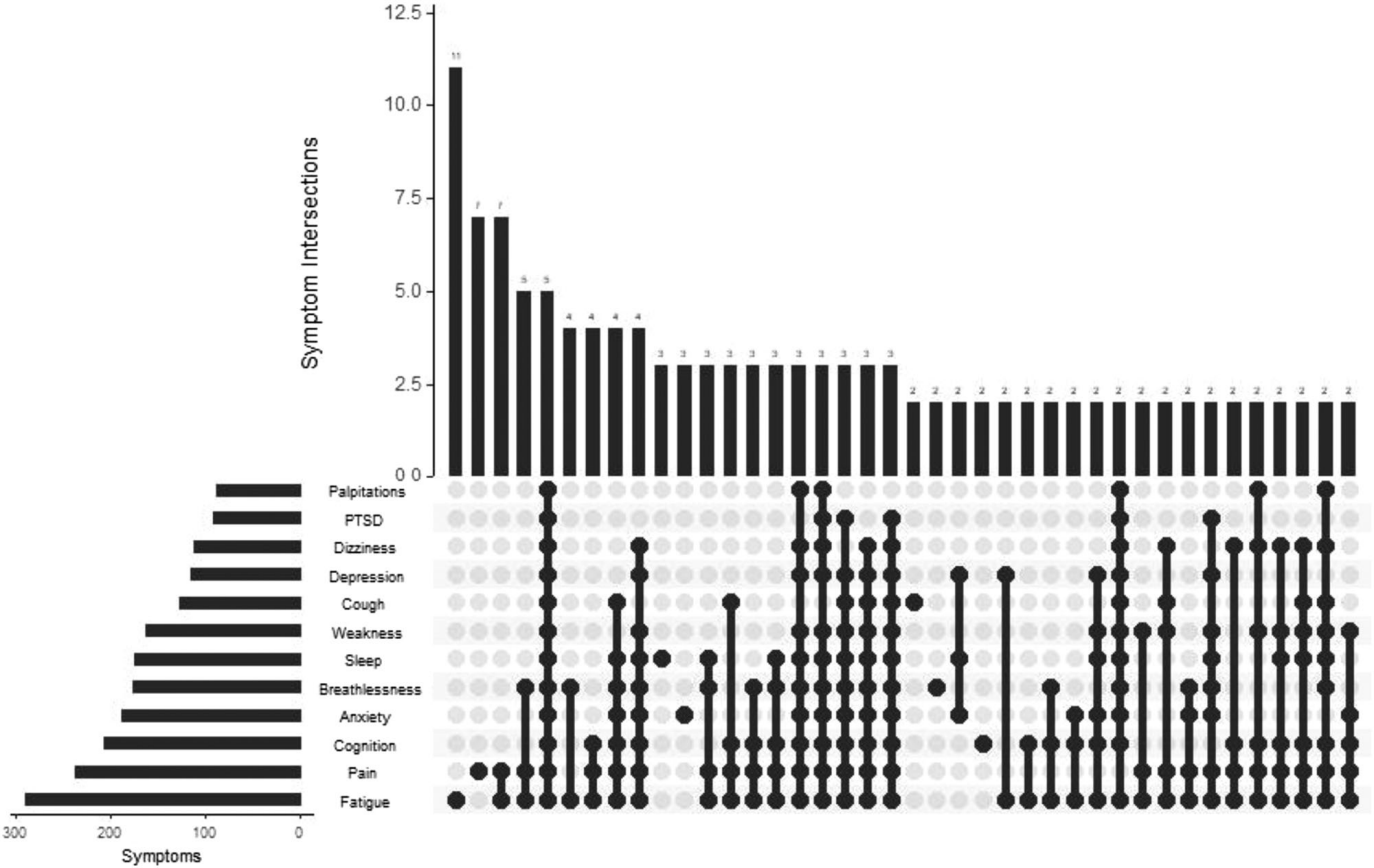
UpSet diagram showing the frequency of different combinations of severe symptoms for Post‐COVID Syndrome

Cluster analysis did not identify any consistent symptom groupings or phenotypes. Dendrograms from average‐linkage hierarchical cluster analysis with Euclidean dissimilarity measures found no distinct separation of subtypes (Figure 5), as indicated by relatively short vertical lines at the top of the dendrogram and evenly spaced dissimilarities across the cohort. This was consistent across different weightings and dissimilarity measures (data not shown). There was substantial overlap between patient symptoms to the extent that applying k‐means cluster analysis with different initial starting values for potential cluster means lead to different categorizations of individuals. The lack of evidence for clustering was also robust to the number of clusters investigated, with cluster means largely defined by the degree of symptom severity across all symptoms, rather than with clustering of distinct types or classes of symptoms.

**Figure 5:**
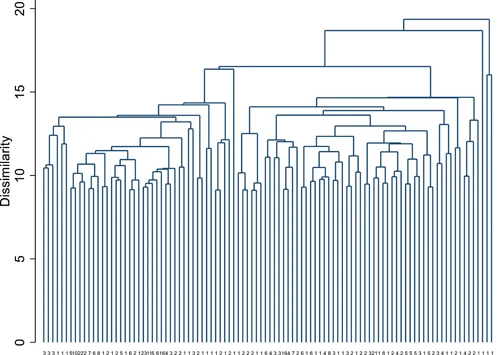
Dendrogram showing the first 100 leaves from agglomerative average linkage cluster analysis. The numbers at the foot of each branch indicate the size of each leaf

## 4 DISCUSSION

This is the first study in the current literature from a community specialist COVID‐19 rehabilitation service reporting on symptom severity phenotypes in a cohort of largely nonhospitalized individuals. We found the severity of a range of individual symptoms was related to the underlying severity of the condition, regardless of hospitalization status during the acute phase of illness. The symptom severity can be clearly categorized into mild, moderate, and severe severity phenotypes. The severity of individual symptoms within each category was similar with a strong correlation between symptoms severity and functional difficulty and moderate correlation between symptom severity and overall health status. Using a range of methods, we did not find evidence for phenotypes based on the type of symptoms.

The severity of individual symptoms within each category being similar might indicate a common underlying pathophysiological mechanism for symptoms in PCS. This also explains the fluctuant nature of the condition where the individual experiences flare‐up of all symptoms (bad days) and symptom‐free days (good days). We did not find any one symptom determining this association or driving other symptoms which are again supportive of underlying common mechanisms in the condition. We have some pointers in the literature towards these common mechanisms in PCS such as vascular damage (hypercoagulability),^17^ immune dysregulation,^18^ and dysautonomia.^19^ The reason for such a heterogeneous presentation of symptoms in individuals needs to be explored in future studies.

There is a concern in PCS that many symptoms such as fatigue or anxiety or mood disorders may already be present in much of the general population before infection. We were however able to show in this study that, for a large number of symptoms, these were not pre‐existing before infection, albeit scored retrospectively. This supports the de novo (new‐onset) nature of the symptoms attributable to the condition of PCS. The data collected in this study also suggests that the C19‐YRS scale can be used to capture pre‐illness symptoms even though there is likely to be a certain degree of recall bias.

Given the growing number of people with PCS (already more than one million in the UK alone), the findings of severity phenotypes in this study could have widespread implications for the provision and resourcing of services to support people living with the condition. The stratification based on the severity of cases could help national and local providers to plan services and interventions that might be directed towards these categories. Mild cases can be investigated in Tier 3 primary care services (such as general practitioners) and offered resources such as Your Covid recovery website^20^ or WHO self‐management booklet.^21^ Moderate cases can be referred to Tier 2 community PCS services where specialist therapy input can be provided. Severe cases need Tier 1 specialist multidisciplinary (MDT) investigations and interventions.^16^, ^22^

One weakness of many previous studies has been the reliance on cohorts entirely comprised of individuals previously hospitalized with COVID‐19. Our inclusion of a large proportion of nonhospitalized patients, and the consistency of their symptoms with those of hospitalized patients, implies that our reported symptoms are unlikely to be a result of the hospital or intensive care experience but are due to the unique underlying pathophysiological mechanism of PCS. This is in keeping with other studies which have shown a similar burden of symptoms in nonhospitalized patients.^23^

It is important to remember that PCS is a fluctuating or episodic condition and that symptom severity can vary over time within the same individual.^24^ Capturing this fluctuation of severity and personal triggers (physical, cognitive, emotional) may help individuals stay within the limits of these triggers and pace their activities accordingly, to avoid worsening of symptom severity and its functional impact. We recommend complex multifaceted rehabilitation interventions to manage symptom severity fluctuation seen in PCS.^16^

There are some limitations to this study. First, a relatively small sample size precludes determining distinct symptom‐based phenotype patterns to be estimated with sufficient precision to be identified. It is possible that rarer phenotypes exist that did not present with sufficient numbers in our study sample. Instead, all groupings of individual symptoms based on correlations between them were dominated by the overall severity across all symptoms. Second, it is worth noting that symptoms and their severity were self‐reported by patients, so there could be a degree of subjectivity in their recording, that they tend to grade severity similar across the symptoms. It is possible that milder cases not presenting to PCS centers for management might present with more distinct symptom clusters that would indicate different underlying pathophysiological mechanisms. Finally, there is an element of recall bias in reporting pre‐illness scores, but this had no bearing on the findings of this study.

## 5 CONCLUSIONS

This is the first study in the current literature reporting on severity phenotypes in a largely nonhospitalized PCS cohort. Severity phenotypes might help stratify patients for targeted interventions and planning of care pathways. Further research is needed to understand the common mechanisms and pathophysiological basis of PCS. Specific symptom‐based phenotype could not be identified in this cohort but needs to be explored in larger population studies.

## CONFLICT OF INTERESTS

The authors declare that there are no conflict of interests.

## AUTHOR CONTRIBUTIONS

Manoj Sivan conceptualized the study, led the development of the C19‐Yorkshire Rehabilitation Scale, and led the writing of the manuscript. Amy Parkin, Sophie Makower, and Manoj Sivan were responsible for the data collection and organization and contributed to the writing of the final drafts of the manuscript. Darren Greenwood is the senior biostatistician in the team and performed all the statistical analyses presented in the draft and contributed to the writing of the manuscript.

## Data Availability

The data sets used and analyzed during the current study are available from the corresponding author on reasonable request.
